# Identifying improved TSPO PET imaging probes through biomathematics: The impact of multiple TSPO binding sites in vivo

**DOI:** 10.1016/j.neuroimage.2011.12.078

**Published:** 2012-04-02

**Authors:** Qi Guo, David R. Owen, Eugenii A. Rabiner, Federico E. Turkheimer, Roger N. Gunn

**Affiliations:** a Division of Experimental Medicine, Imperial College London, Hammersmith Hospital, UK; b GlaxoSmithKline Clinical Imaging Centre, Hammersmith Hospital, London, UK; c Department of Engineering Science, University of Oxford, UK

**Keywords:** TSPO, PET, Radioligand, Biomathematical modelling, Multiple binding affinities

## Abstract

To date, ^11^C-(R)-PK11195 has been the most widely used TSPO PET imaging probe, although it suffers from high non-specific binding and low signal to noise. A significant number of 2nd generation TSPO radioligands have been developed with higher affinity and/or lower non-specific binding, however there is substantial inter-subject variation in their affinity for the TSPO. TSPO from human tissue samples binds 2nd generation TSPO radioligands with either high affinity (high affinity binders, HABs), or low affinity (LABs) or expresses both HAB and LAB binding sites (mixed affinity binders, MABs). The expression of these different TSPO binding sites in human is encoded by the rs6971 polymorphism in the TSPO gene. Here, we use a predictive biomathematical model to estimate the in vivo performances of three of these 2nd generation radioligands (^18^F-PBR111, ^11^C-PBR28, ^11^C-DPA713) and ^11^C-(R)-PK11195 in humans. The biomathematical model only relies on in silico, in vitro and genetic data (polymorphism frequencies in different ethnic groups) to predict the radioactivity time course in vivo. In particular, we provide estimates of the performances of these ligands in within-subject (e.g. longitudinal studies) and between-subject (e.g. disease characterisation) PET studies, with and without knowledge of the TSPO binding class. This enables an assessment of the different radioligands prior to radiolabelling or acquisition of any in vivo data.

The within-subject performance was characterised in terms of the reproducibility of the in vivo binding potential (%*COV*[*BP*_*ND*_]) for each separate TSPO binding class in normal and diseased states (50% to 400% increase in TSPO density), whilst the between-subject performance was characterised in terms of the number of subjects required to distinguish between different populations.

The results indicated that the within-subject variability for ^18^F-PBR111, ^11^C-PBR28 and ^11^C-DPA713 (0.9% to 2.2%) was significantly lower than ^11^C-(R)-PK11195 (16% to 36%) for HABs and MABs in both normal and diseased states. For between-subject studies, sample sizes required to detect 50% differences in TSPO density with the 2nd generation tracers are approximately half that required with ^11^C-(R)-PK11195 when binding class information is known a priori. As binding class can be identified using a simple genetic test or from peripheral blood assays, the combination of binding class information with 2nd generation TSPO imaging data should provide superior tools to investigate inflammatory processes in humans in vivo.

## Highlights

► Biomathematics is used to predict the in vivo performance of TSPO PET tracers. ► Multiple binding affinities of the TSPO tracers are accounted for. ► Polymorphism frequencies in different ethnic groups are considered. ► 2nd generation TSPO tracers are predicted to be better than ^11^C-(R)-PK11195. ► Improvements are predicted for both within and between subject studies.

## Introduction

Neuroinflammation plays an important role in neurodegenerative disorders, and thus there is a demand for reliable imaging markers of inflammatory processes that can act as diagnostic tools and support the development of new drugs. Positron Emission Tomography (PET) has the unique ability to provide quantitative information of active inflammatory processes by targeting the translocator protein (TSPO, formerly known as the peripheral benzodiazepine receptor). The TSPO has been observed in higher density in activated macrophages and microglia across various brain diseases (Banati, 2002).

TSPO expression has been imaged in the central nervous system (CNS) using the selective antagonist PK11195 labelled with the positron emitting radionuclide C-11 for more than 15 years (Cagnin et al., 2007; Shah et al., 1994). Imaging of neuroinflammatory processes has been demonstrated with ^11^C-(R)-PK11195 in traumatic, inflammatory, degenerative, and neoplastic diseases (Cagnin et al., 2007). However, the high non-specific binding of ^11^C-(R)-PK11195, its low extraction in brain and the resulting poor signal-to-noise ratio of ^11^C-(R)-PK11195 have led to the need for improved TSPO PET radioligands. In the past 5 years, more than 50 candidate 2nd generation TSPO ligands have been introduced, with some offering higher specific to non-specific signals including ^18^F-FEPPA, ^18^F-PBR111, ^11^C-PBR28, ^11^C-DPA713, ^11^C-DAA1106, and ^11^C-AC-5126 (Chauveau et al., 2008).

However, the use of these 2nd generation TSPO radioligands has been complicated by the existence of apparent “non-binders” that were identified in approximately 10% of humans scanned with the prototypical radioligand, ^11^C-PBR28 (Brown et al., 2007; Fujita et al., 2008; Kreisl et al., 2010). These subjects appeared not to bind PBR28. In vitro radioligand binding studies using post-mortem human brain tissue revealed substantial population variation in the affinity of PBR28 for the TSPO. “Low affinity binders” (LABs) have a marked reduction in affinity for PBR28 (*K*_*i*_ ~ 188 nM) in comparison to “high affinity binders” (HABs, *K*_*i*_ ~ 3.4 nM). Hence LABs do not produce a measurable specific signal in PET studies with ^11^C-PBR28 and appear as “non-binders” (Owen et al., 2010). A third group was also identified (“mixed affinity binders” (MABs)), who express the HAB and LAB binding sites in approximately equal number (Owen et al., 2010). This variation in binding affinity is not specific to PBR28; all TSPO radioligands tested behave in a similar manner, but for PK11195 which appears to bind with similar affinity in all subjects (Owen et al., 2011). This variation in binding affinity is due to a single nucleotide polymorphism (rs6971) in the gene encoding the TSPO (Owen et al., 2012), which causes a substitution of threonine and alanine at position 147 (Ala147Thr). This substitution is common in Caucasians: 49% of subjects are Ala-Ala (HABs), 42% are Ala-Thr (MABs), and 9% are Thr-Thr (LABs), but its prevalence varies across ethnic groups.

The existence of HABs, MABs and LABs complicates the interpretation of the specific binding signal, as the binding signal of a particular TSPO ligand depends on its ratio of affinities for the high- and low-affinity sites, and the binding class of the individual. Therefore measured differences in vivo could simply result from a difference in affinity (binding class) rather than TSPO density. Since the binding class is consistent within each subject for all the 2nd generation ligands tested to date, genotyping the rs6971 polymorphism can help the quantitative assessment of TSPO ligand binding, by allowing binding affinity to be predicted at screening.

The aim of this work is to use predictive biomathematical models to investigate the performance and utility of some of the most promising 2nd generation TSPO radioligands, namely ^18^F-PBR111, ^11^C-PBR28, and ^11^C-DPA713, in both within-subject and between-subject studies, taking into account multiple binding classes, and compare their quantitative performance with ^11^C-(R)-PK11195. These predictive biomathematical models allow for a prediction of the in vivo performance simply by using appropriate in silico and in vitro data and have been validated previously for a wide range of ligands that do not involve genetic variability (Guo et al., 2009). In this work, we extend the previous approach to account for a genetic dependency of the signal which is a consequence of the multiple TSPO binding classes and affinities whose frequencies can also differ across ethnic groups. This approach facilitates the identification of optimal TSPO PET tracers prior to the acquisition of any in vivo data. For example, we evaluate in longitudinal studies whether these radioligands are likely to be more sensitive to detect fluctuations in inflammation processes (fewer number of subjects required for equivalent power) than ^11^C-(R)-PK11195 for each of the binding classes, and whether these 2nd generation radioligands are superior to ^11^C-(R)-PK11195 in between-subject studies designed to compare healthy subjects to those with a particular disease, with and without knowledge of their binding classes.

## Materials and methods

### The outline of the biomathematical framework

**Fig. 1:**
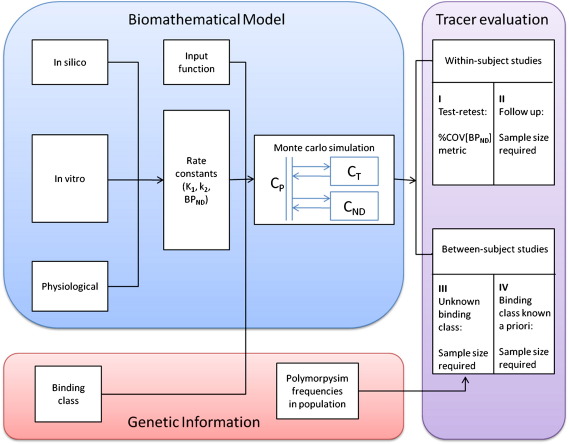
The framework of the biomathematical modelling approach to evaluate the performances of the TSPO ligands.

The outline of the biomathematical framework is shown in Fig. 1 and is based on approximating the tracer's behaviour by a single tissue compartment model (1TCM). In silico, in vitro data, and physiological parameters were used to predict the influx (*K*_1_), efflux rate constants (*k*_2_) as well as the binding potential (*BP*_*ND*_, defined as the ratio of specifically bound to nondisplaceable radioligand in tissue, (Innis et al., 2007)) for the compartmental structure. In order to account for the multiple affinities, binding class status was used to determine the appropriate specific signal for different individuals. Monte Carlo simulations were used to generate a large population of *BP*_*ND*_ values for groups in healthy and diseased states with different TSPO levels. For within-subject studies, the numerical identifiability of the ligands *BP*_*ND*_ were evaluated and sample sizes required for follow up studies were estimated. For between-subject studies, with additional information on the frequency of different binding classes in an ethnic group (Owen et al., 2012), we investigated the power of the different radioligands to distinguish diseased from healthy subjects in terms of the minimal sample sizes required with and without knowledge of the binding class status of each subject.

### The in silico/in vitro data

The in silico and in vitro data required by the biomathematical models to predict the in vivo radioligand performance include the lipophilicity (*clogD*), McGowan molecular volume (*Vx*), free fractions in plasma and tissue (*f*_*p*_ and *f*_*ND*_), target density (*B*_*max*_) and affinity represented by *K*_*D*_ or *K*_*i*_ (Guo et al., 2009).

Here, the *clogD* and *Vx* of ^18^F-PBR111, ^11^C-PBR28, ^11^C-DPA713 and ^11^C-(R)-PK11195 were determined using in-house in silico models based on the structures of the compounds. The *f*_*P*_ and *f*_*ND*_ were measured from Landrace pig tissue (blood and brain — obtained from Charles River Laboratories, Tranent, Scotland) using equilibrium dialysis (Summerfield et al., 2006). In brief, the cold test compounds were spiked into plasma or brain homogenate at a concentration of 1 μg/g and dialysed against phosphate buffered saline (PBS, pH 7.4) for 5 h at 37 °C. Aliquots (20–50 μL) of plasma, brain and dialysate were taken and extracted using acetonitrile containing internal standard, followed by LC–MS/MS analysis. The free fractions were determined as the concentration ratio of analyte in dialysate to that in plasma or brain. The density of TSPO in different binding classes and affinities of these compounds was obtained from literature (Owen et al., 2010, 2011). The *B*_*max*_ was measured in post mortem brain samples in cortex (with no evidence of inflammation on immunohistochemistry) obtained from UK MS tissue bank using homogenate saturation binding assays at 37 °C with ^3^H-PK11195 (purchased from Perkin Elmer, Cambridge, UK) and ^3^H-PBR28 (custom labelled by GE Healthcare, Amersham, UK) respectively (Owen et al., 2010). Affinity (*K*_*i*_) of PBR111, DPA713, PBR28, and PK11195 was measured using competition assays for each binding class with ^3^H-PK11195 at 37 °C (Owen et al., 2011).

### The biomathematical modelling approach

A biomathematical modelling approach was used to predict the in vivo performance of the radioligands whilst accounting for multiple binding classes (Fig. 1). The use of the approach to predict how good a radioligand is for a single target has been described in detail previously (Guo et al., 2009). In brief, it uses a 1TCM to approximate both the non-displaceable and total uptake of a radioligand. The 1TCM is described by a parsimonious parameter set including the influx rate constant *K*_1_, the efflux rate constant of the non-displaceable tissue *k*_2_, and the binding potential *BP*_*ND*_, all of which can be predicted from the in silico and in vitro data. The approach assumes that the radioligands cross the blood brain barrier (BBB) by passive diffusion and yields equations for *K*_1_ and *k*_2_ as:

(1) $$K_{1}=f\;\left(1-e^{-\frac{PS\;}{f}}\right)$$

where

(2) $$P=10^{-0.121\;{\left(c\log D-2.298\right)}^{\;2}-2.544\log\left(Vx{\;}^{1/3}\right)-2.525}$$

(3) $$k_{2}=\frac{V_{aq\_p}\;K_{1}}{V_{aq\_T}}\;\frac{f_{\mathrm{ND}}}{f_{p}}$$

where *f* is perfusion in units of mL·cm^−3^·min^−1^, *P* is the permeability of the compound in cm·min^−1^, *S* is the capillary surface area in cm^2^·cm^−3^, *V*_*aq_P*_ and *V*_*aq_T*_ are the apparent aqueous volume to correct for the small volume that cannot be reached by solvent in plasma and tissue respectively. In this paper, the following values were used for these physiological parameters: *f* = 0.6 mL·cm^−3^·min^−1^, *S* = 150 cm^2^.cm^−3^ brain, *V*_*aq_P*_ = 0.98 solvent/mL plasma and *V*_*aq_T*_ = 0.9 solvent/mL tissue. In addition, a scaling factor of 3.43 was introduced to account for in vitro–in vivo differences in *K*_1_ (Guo et al., 2009).

*BP*_*ND*_ can be predicted from *f*_*ND*_, *B*_*max*_ and *K*_*D*_ for a single target. Here, we extended the model to account for multiple TSPO binding classes by considering the different affinities for binding of the radioligands. *BP*_*ND*_ was predicted depending on the binding class status (HABs, MABs and LABs) as:

(4) $$BP_{\mathrm{ND}}\begin{array}{cc} =f_{\mathrm{ND}}\frac{B_{\max}{}_{H}}{K_{D}{}_{H}} & \text{for HABs}\\ =f_{\mathrm{ND}}\frac{B_{\max}{}_{\mathrm{MH}}}{K_{D}{}_{H}}+f_{\mathrm{ND}}\frac{B_{\max}{}_{\mathrm{ML}}}{K_{D}{}_{L}} & \text{for MABs}\\ =f_{\mathrm{ND}}\frac{B_{\max}{}_{L}}{K_{D}{}_{L}} & \text{ for LABs} \end{array}$$

where *K*_*DH*_ and *K*_*DL*_ are the high and low affinities for TSPO for each ligand, *B*_*maxH*_ and *B*_*maxL*_ are the density of TSPO in HABs, LABs, and *B*_*maxMH*_ and *B*_*maxML*_ are the density of high affinity sites and low affinity sites in MABs. Here, *K*_*i*_ values were used instead of *K*_*D*_ for all the compounds considered to be consistent.

Using these parameters, time activity curves (TACs) were generated for both target and non-displaceable tissue using a standard plasma input function as follows:

(5) $$C_{T}(t)=\left(1-V_{B}\right)K_{1}e^{-\frac{k_{2}}{1+BP_{\mathrm{ND}}}t}*C_{P}(t)+V_{B}C_{B}(t)$$

(6) $$C_{\mathrm{ND}}(t)=\left(1-V_{B}\right)K_{1}e^{-k_{2}t}*C_{P}(t)+V_{B}C_{B}(t)$$

where *C*_*T*_(*t*) and *C*_*ND*_(*t*) are the concentration of the radioligands in total tissue and non-displaceable compartment respectively; *C*_*P*_(*t*) and *C*_*B*_(*t*) are the concentration in plasma and whole blood respectively, and *V*_*B*_ is the blood volume. Here the *V*_*B*_ was fixed to 5% in the estimation, and we used an ^11^C-(R)-PK11195 human input function as a standard input for all the radioligands. The validity of using a standard input function has been verified in Guo et al. (2009).

### Within-subject performance assessment

The within-subject performance of the radioligands was assessed in terms of the numerical identifiability of the specific signal (*BP*_*ND*_) and the power to monitor disease progression in longitudinal studies. Here, the *BP*_*ND*_ was chosen as the primary outcome measure of interest, and the identifiability of the *BP*_*ND*_ was evaluated in terms of its coefficient of variation (%*COV*[*BP*_*ND*_]), which can be estimated from Monte Carlo simulations.

In order to estimate the %*COV*[*BP*_*ND*_], the *K*_1_, *k*_2_ and *BP*_*ND*_ predicted from in silico/in vitro data were used in conjunction with the 1TCM to generate noiseless TACs (for 90 min) as shown in Eqs. 5 and 6. Subsequently, 1000 noisy realisations were generated with noise added to the non-decay corrected data. The variance of the noise was proportional to the non-decay corrected radioactivity/frame duration, and the scale of noise was determined based on experiences with the noise level found in actual in vivo ROI based TACs. The simulated TACs with noise were then decay corrected considering different isotopes (Logan et al., 2011) and the 1TCM was fitted to both the non-displaceable and the total tissue data to derive estimates of *BP*_*ND*_ as *BP*_*ND*_ = *V*_*T*_ / *V*_*ND*_ − 1, where *V*_*T*_ and *V*_*ND*_ are the volumes of distribution estimated from *K*_1_/*k*_2_ in the total and non-displaceable compartments respectively. This Monte Carlo simulation then allowed for the calculation of %*COV*[*BP*_*ND*_]:

(7) $$\%COV[BP_{\mathrm{ND}}]=\frac{\sigma (BP_{\mathrm{ND}})}{\mu (BP_{\mathrm{ND}})}\times 100\%$$

where *σ*(*BP*_*ND*_) is the standard deviation and *μ*(*BP*_*ND*_) is the mean of the *BP*_*ND*_ estimates derived from the simulations. This metric was used to assess radioligand performance, with a smaller %*COV*[*BP*_*ND*_] value indicating a better radioligand.

A %*COV*[*BP*_*ND*_] was produced for each binding class with each radioligand. In order to estimate the identifiability of the *BP*_*ND*_ for a diseased population, the *B*_*max*_ of TSPO for all the binding classes was increased by 50% and 400% to approximate a moderate and high increase of TSPO, and the simulations were performed to estimate the %*COV*[*BP*_*ND*_] accordingly. 50% and 400% were chosen to represent diseases with various levels of TSPO increase, i.e. about 50% increase as found in schizophrenia (Doorduin et al., 2009) and about 400% in amyotrophic lateral sclerosis (ALS) (Venneti, et al., 2008). Note that there will be some inter-subject variability for the increase in binding. However, as the exact levels of TSPO changes are not known precisely for different diseases, here we fixed the increase level to 50% and 400% as a first order approximation to identify whether these 2nd generation ligands are improvements over ^11^C-(R)-PK11195.

In addition, to evaluate the capabilities of the radioligands to monitor disease progression, we performed power analyses to estimate the sample size required with each radioligand to detect 50% or 400% change in the TSPO density within subject. A population of 1000 subjects was simulated for each binding class separately (HABs, MABs and LABs) using the biomathematical modelling approach with 10% variation in *K*_1_ and *k*_2_ and 25% in *B*_*max*_ to account for the variability in the population. For each subject, TACs were generated with *B*_*max*_ at a normal value as well as at both diseased levels with fixed values for *K*_1_ and *k*_2_. *BP*_*ND*_ was estimated for all the realisations of these three TACs for each binding class. The mean and the variance of *BP*_*ND*_ were then calculated from each group (HABs, MABs and LABs) in the population, and the sample size required for 90% power was assessed using a paired *t*-test with α = 0.05.

### Between-subject performance assessment

The multiple binding classes introduce a higher complexity for interpreting the differences in binding signals between subjects. Here, we aim to evaluate the capability of the ligands to distinguish between groups of diseased and healthy subjects when binding class status is either known or unknown. For the scenario when the binding class status is not available, a population of 1000 subjects containing all binding classes was simulated based on the biomathematical modelling approach. Since there is a difference in the frequencies of the rs6971 polymorphism in different ethnic groups, namely Caucasians, African Americans, Han Chinese and Japanese, we evaluated each group separately. The proportion of each binding class in the Caucasian population used for simulation is HAB:MAB:LAB = 49:42:9, and 56:38:6 for African Americans and 94:6:0.001 for Chinese and Japanese (Owen et al., 2012), and these proportions are fixed according to the polymorphism frequency in each ethnic group. The percentage of HABs, MABs and LABs were assumed to be the same in the control group and in the pathological cohort. Within each population, the variability for different subjects was considered by adding 10% variability to *K*_1_, 20% to *k*_2_, and within each binding class, 25% to *B*_*max*_. Mean and variance of the *BP*_*ND*_ of the healthy and diseased groups were estimated from the total population.

Power analyses were performed to calculate the sample size required to detect a 50% or 400% difference between the two groups (0.9 power using two sample *t*-test with different variances). Note that when different binding classes are mixed together, the distribution of the total population is not normal any more. A Mann–Whitney *U* test was used for the power calculation for the mixed population rather than *t*-test. The sample size required for a *U* test was estimated by a correction of the Pitman Asymptotic Relative Efficiency (A.R.E.) to the *t*-test sample size (Randles and Wolfe, 1979).

In addition, sample sizes were also calculated under the assumption that the binding class information is known a priori. In this case, subjects can be separated into different binding classes, which allows for a comparison of healthy and diseased groups within each binding class. A two sample *t*-test was used for these analyses.

## Results

### Estimation of the in silico and in vitro parameters

The in silico/in vitro data required by the biomathematical models to predict the in vivo parameters are listed in Table 1. In contrast to ^11^C-(R)-PK11195 which has a very small free fraction in tissue (*f*_*ND*_ = 0.01), all the 2nd generation radioligands have a much higher *f*_*ND*_, indicating much lower non-specific binding in tissue. In addition, the new ligands have higher affinities for HABs than ^11^C-(R)-PK11195 does, although with significantly lower affinities for LABs. ^11^C-(R)-PK11195 displayed similar affinity for both HABs and LABs with an affinity ratio indistinguishable from 1 (0.8; Owen et al., 2011). *B*_*max*_ values for HABs, MABs, and LABs measured in a homogenate saturation assay using ^3^H-PBR28 were used for ^18^F-PBR111 and ^11^C-DPA713 as well. The *B*_*max*_ values for ^11^C-(R)-PK11195 were measured separately using a homogenate saturation assay and ^3^H-PK11195. Here we assumed that the TSPO density of both the high and low affinity sites in MABs is the same (*B*_*max-MH*_ = *B*_*max-ML*_ = 50% *B*_*max-M*_; Owen et al., 2010).

### Comparison of the radioligand performance in within-subject studies

The predicted in vivo *K*_1_, *k*_2_ and *BP*_*ND*_ values are listed in Table 2. ^11^C-(R)-PK11195 shows lower brain entry compared to the other ligands represented by a smaller *K*_1_. For both HABs and MABs, the new ligands have 2 to 20 fold higher *BP*_*ND*_ values as compared to ^11^C-(R)-PK11195.

**Fig. 2:**
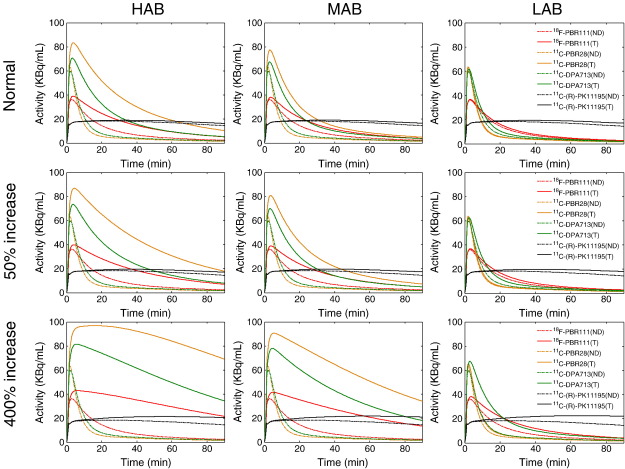
Prediction of the TACs for each radioligand for all the binding classes in both normal and diseased states with moderate (50% increase) and high (400% increase) levels of TSPO. T: total tissue; ND: nondisplaceable tissue.

Time activity curves were predicted for each radioligand, for each binding class, at both normal and diseased states (Fig. 2). In general, the new radioligands demonstrate a much higher uptake and specific to non-specific binding ratio as compared to ^11^C-(R)-PK11195 for HABs and MABs in all conditions. For LABs, at normal and moderate TSPO levels, the new ligands produced lower specific binding signal similar to ^11^C-(R)-PK11195. This is consistent with the overall prediction results represented by %*COV*[*BP*_*ND*_] (see Table 3). The model predicted small %*COV*[*BP*_*ND*_] values for all the new ligands (<5%) for both HABs and MABs at all states whereas the %*COV*[*BP*_*ND*_] of ^11^C-(R)-PK11195 is higher than 15% in all scenarios. For LABs, all the other 2nd generation ligands showed a much smaller %*COV*[*BP*_*ND*_] at all states except ^11^C-PBR28 which had a significantly lower affinity for low affinity binding sites. However, when there are high levels of TSPO, the %*COV*[*BP*_*ND*_] of ^11^C-PBR28 for LABs reduced to under 10%, which was much smaller than ^11^C-(R)-PK11195 (17%).

The sample sizes required for longitudinal studies within subjects using these ligands were consistent with their %*COV*[*BP*_*ND*_] estimates (Table 4). For HABs and MABs, it required 3 to 4 subjects in the control and diseased groups to identify a 50% change in TSPO density (90% power) except ^11^C-PBR28 for LABs, whilst ^11^C-(R)-PK11195 requires over 15 subjects in each group. To identify a 400% increase, the sample size required for all ligands was similar with around 3 to 4 subjects required in each group.

### Comparison of the radioligand performance in between-subject studies

When no information is available on binding class a priori, even when all of the subjects are healthy, the variance of the *BP*_*ND*_ values can be very large depending on which radioligand is used and its high:low binding site affinity ratio.

**Fig. 3:**
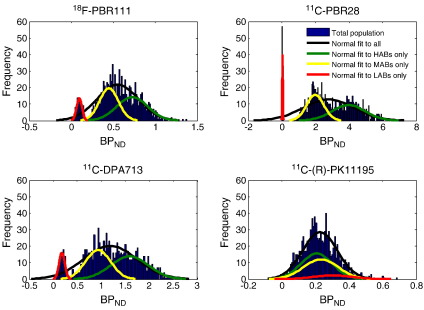
Predicted *BP*_*ND*_ distributions for ^18^F-PBR111, ^11^C-PBR28, ^11^C-DPA713 and ^11^C-(R)-PK11195 from a population of 1000 healthy Caucasians consisting of HABs, MABs and LABs.

We simulated the binding of a population of 1000 healthy controls from different binding classes, and an example of *BP*_*ND*_ histograms of Caucasians is shown in Fig. 3. Also we fitted the histogram of each individual binding class in the population separately. The plot shows that for ^18^F-PBR111, ^11^C-PBR28 and ^11^C-DPA713, the distribution of the *BP*_*ND*_ appears to be tri-normal rather than the normal behaviour observed for ^11^C-(R)-PK11195. This is due to the large affinity ratios for the three new ligands (~4 for ^18^F-PBR111, ~55 for ^11^C-PBR28 and ~4 for ^11^C-DPA713 compared to ^11^C-(R)-PK11195, which has an affinity ratio of approximately 1) and is consistent with the three binding classes.

The multiple affinities of these new ligands for different subjects lead to a large variance of the binding potential within a population, which would further require more samples to detect a difference between the healthy controls and the diseased subjects. However, the sample size required for between-subject studies is also related to the reproducibility of the ligand itself, in other words, the within-subject variability of the radioligand.

The sample sizes required to detect 50% and 400% increase of TSPO in studies comparing different populations when the binding class is unknown for all the ethnic groups considered here are listed in Table 5. It shows that the sample sizes required by the 2nd generation ligands to characterise a 50% change in TSPO are higher (33 to 49) compared to ^11^C-(R)-PK11195 (~29) in Caucasians. Fewer samples (~4 to 8) are needed to characterise a 400% change with all radioligands. In African Americans, similar performances of all ligands were predicted (sample size ~29) except ^11^C-PBR28, which requires more samples to detect 50% increase in TSPO density. In Han Chinese and Japanese, the 2nd generation ligands significantly outperformed ^11^C-(R)-PK11195 by reducing sample size to detect 50% density difference by half.

We also estimated the sample size required if the binding class information was known a priori (Table 6). In this case, subjects in the control group and the diseased group were divided into different binding classes and compared within their own class. The sample sizes required for the new ligands to detect 50% change reduced to about 10 subjects except ^11^C-PBR28 for LABs (which requires 22 subjects), whilst ^11^C-(R)-PK11195 still requires more than 20 subjects.

## Discussion

In this study, the in vivo performance of three 2nd generation TSPO ligands (namely ^18^F-PBR111, ^11^C-PBR28, and ^11^C-DPA713) and ^11^C-(R)-PK11195 was predicted directly from in silico/in vitro data using a biomathematical modelling approach (Guo et al., 2009) which has been extended here to account for tracers with multiple binding sites. These radioligands were chosen because the in vitro data required by the models had been acquired previously. The radioligands were compared in terms of their performances in within-subject studies and between-subject studies based on this modelling approach. The models predict that the 2nd generation TSPO radioligands are better ligands than ^11^C-(R)-PK11195 for both longitudinal and disease characterisation studies that take into account the binding status.

This process of predicting in vivo behaviour from in silico and in vitro data is complex and rests on a number of modelling assumptions which are important to consider. The method uses a 1TC model to characterise the tissue kinetics of the radioligands under the assumption that the radioligand equilibrates rapidly between all tissue compartments. Though this may not always be the case and in practice, the in vivo data may be analysed by other quantification methods rather than 1TC, given the overall complexity of in vivo prediction directly from in silico and in vitro data, the assumption was reasonable and allowed for a parsimonious description. It has been shown previously that a wide range of tracers and binding levels can be successfully approximated by a 1TC in the context of predicting the performance of radioligands via the %COV of *BP*_*ND*_ (Guo et al., 2009). The relative power of the tracers will to a large extent hold true regardless of the quantification method used, as the main driven factors are the characteristics of the chemical compounds. Here, a plasma input function obtained from a previous human ^11^C-(R)-PK11195 study was used as a standard input function for all the radioligands and TACs of 90 min duration were simulated. Although the pharmacokinetics of the ligands might be different and longer scanning time might be optimal for some of the ligands such as ^18^F-PBR111, we had found previously that small variations in the input function do not have a significant impact on the overall prediction and this is a consequence of the outcome measure of interest, *BP*_*ND*_, being a ratio of two distribution volumes (*V*_*T*_ and *V*_*ND*_) which rely on the same input (Guo et al., 2009). The estimates of the plasma and tissue free fractions were measured in pig tissue using equilibrium dialysis. *f*_*ND*_ has been shown to be consistent across species (Summerfield et al., 2008) although there could be species differences in *f*_*P*_. Larger *f*_*p*_ values measured in human blood using ultrafiltration have been shown in Fujita et al. (2008) and Endres et al. (2009). More understanding is required around differences between ultrafiltration and dialysis measurements particularly at low concentrations. In addition, the measurement of other parameters in vitro, such as the affinity of the radioligands for TSPO, may differ from the actual values in vivo due to assay conditions or differences in the tissue environment in vivo.

However, in general, the models generated reasonable predictions for the radioligands. In the literature, the in vivo *BP*_*ND*_ value for ^11^C-(R)-PK11195 in healthy brain is around 0.23–0.45 (Turkheimer et al., 2007), and the model prediction is around 0.2. The in vivo *K*_1_ values in the literature for ^11^C-PBR28 (0.10–0.14 mL/cm^3^/min) and ^11^C-DPA713 (0.20–0.29 mL/cm^3^/min) are higher than ^11^C-(R)-PK11195 (0.016–0.026 mL/cm^3^/min) (Endres et al., 2009; Fujita et al., 2008). This is consistent with our prediction which indicated that the 2nd generation TSPO ligands considered here enter the brain well with *K*_1_ values higher (0.25–0.48 mL/cm^3^/min) than that of ^11^C-(R)-PK11195 (0.09 mL/cm^3^/min). In addition, as *f*_*ND*_ is an index of non-specific binding, the much higher values for the 2nd generation ligands are consistent with them having lower non-specific binding as compared to ^11^C-(R)-PK11195.

Overall, the models predicted that the new radioligands perform better in vivo than ^11^C-(R)-PK11195 for HABs and MABs in both normal and diseased states as represented by smaller %*COV*[*BP*_*ND*_] values. For longitudinal studies, the models predicted that the new ligands have better numerical identifiability than ^11^C-(R)-PK11195, and as a result, fewer subjects would be required to detect moderate TSPO increase. When considering variability between subjects in addition to the variability within subject, because of the different affinities of the radioligands for different binding sites, the distribution of a mixed population of subjects from all the binding groups is not normal any more. We simulated the possible distribution of the binding potentials within a population and it demonstrated the same pattern as the distribution of the V_T_ values observed in vivo in a previous study using ^11^C-PBR28 (Owen et al., 2011). For between-subject studies when subjects from different binding classes are considered together, power analyses were performed to estimate the sample sizes required for the ligands to distinguish diseased from healthy subjects. The models predicted that the effect size for ^11^C-(R)-PK11195 to identify a 50% difference in TSPO level is about 1, which is consistent with in vivo *findings* such as in Tomasi et al. (2008), where ^11^C-(R)-PK11195 was used to characterise Alzheimer's disease.

Note that due to the different rs6971 polymorphism frequencies in the ethnic groups considered here in between-subject studies, when the binding class information is not known, the performance of the 2nd generation ligands is significantly better than ^11^C-(R)-PK11195 in Chinese and Japanese, similar in African Americans and slightly less competent in Caucasians. This indicates that caution is needed when the 2nd generation ligands are used in Caucasians because of the existence of relatively large proportion of LABs.

**Fig. 4:**
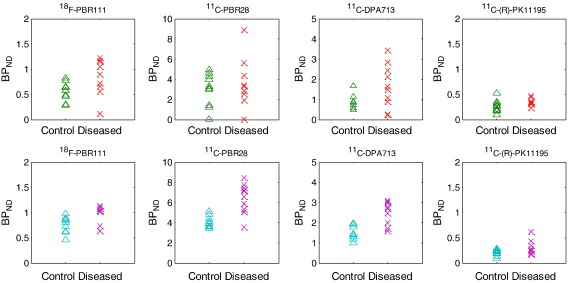
Simulations of 10 random samples from a population of mixed subjects and HABs only at normal and moderate TSPO increase states (50%). Top row: mixed subjects from different binding classes; bottom row: HABs only.

However, if we know the binding class information of subjects a priori, and are able to stratify subjects and compare them within the same binding class, the 2nd generation ligands require much fewer samples to detect a moderate TSPO increase. Fig. 4 shows simulations of 10 random samples from a population of either the full population or HABs only at normal and 50% TSPO increase states. This figure demonstrates the superiority of the 2nd generation ligands when there is a priori knowledge of the binding class of each subject. When only HABs are included for example, there is clearer separation between the control and diseased groups with higher *BP*_*ND*_ compared to ^11^C-(R)-PK11195.

Recent work conducted by Owen and colleagues now makes identification of the binding class of individuals feasible from either a genetic test or a peripheral blood assay. This allows TSPO binding class to be determined by a simple genetic test prior to quantitative PET scans of TSPO expression, and gives increased value and confidence in using the 2nd generation TSPO radioligands.

In general, the predicted performances of all the 2nd generation tracers examined were roughly similar with each other, with ^11^C-DPA713 and ^18^F-PBR111showing higher numerical identifiability within all binding classes whilst ^11^C-PBR28 was not very promising for LABs. However, these differences were modest and below the level of confidence provided by the biomathematical modelling approach, In vivo human PET studies will help clarify a frontrunner amongst the 2nd generation TSPO PET probes.

## Conclusions

The biomathematical modelling approach is a useful tool to predict and compare the in vivo performance of TSPO radioligands directly from in silico/in vitro data prior to intensive radiochemistry and preclinical/clinical experimentation. The approach considers brain entry, specific/non-specific binding and kinetics as well as multiple affinity binding to two sites. The models predict that the 2nd generation TSPO radioligands considered here, namely ^18^F-PBR111, ^11^C-PBR28 and ^11^C-DPA713, are better than ^11^C-(R)-PK11195 for both longitudinal and disease characterisation studies if binding class information is known a priori. Combined with recent work that allows binding class to be identified from a single polymorphism (rs6971) in the TSPO gene or a platelet assay, this demonstrates the great potential of using 2nd generation TSPO radioligands for PET studies of neuroinflammation.

## Figures and Tables

**Table 1:** In silico and in vitro properties of radioligands.

Radioligands	In silico		In vitro equilibrium dialysis		In vitro homogenate binding				
	*Vx*	*clogD*	*f*_*p*_	*f*_*ND*_	*B*_*max-H*_	*B*_*max-M*_^a^	*B*_*max-L*_	*K*_*i-H*_	*K*_*i-L*_
^18^F-PBR111	3.10	4.0	0.19 ± 0.018 (n = 6)	0.06 ± 0.005 (n = 6)	193 ± 66 (n = 6)	186 ± 37 (n = 4)	99.4 ± 35 (n = 5)	15.6 ± 3.7 (n = 5)	61.8 ± 10.7 (n = 5)
^11^C-PBR28	2.69	2.8	0.16 ± 0.044 (n = 6)	0.07 ± 0.006 (n = 6)	193 ± 66 (n = 6)	186 ± 37 (n = 4)	99.4 ± 35 (n = 5)	3.40 ± 0.2 (n = 6)	188 ± 7.0 (n = 5)
^11^C-DPA713	2.91	3.0	0.33 ± 0.034 (n = 6)	0.12 ± 0.006 (n = 6)	193 ± 66 (n = 6)	186 ± 37 (n = 4)	99.4 ± 35 (n = 5)	15.0 ± 2.2 (n = 5)	66.4 ± 7.8 (n = 4)
^11^C-(R)-PK11195	2.74	4.9	0.14 ± 0.039 (n = 4)	0.01 ± 0.002 (n = 6)	433 ± 154 (n = 9)	433 ± 154^b^ (n = 9)	506 ± 131 (n = 5)	28.3 ± 4.0 (n = 6)	22.3 ± 2.2 (n = 5)

Units for *Vx* is cm^3^ mol^−1^/100; units for *B*_*max*_ and *K*_*i*_ are nM; *clogD*, *f*_*P*_ and *f*_*ND*_ are unitless. *B*_*max*_ and *K*_*i*_ values were obtained from Owen et al. (2010, 2011).

a *B*_*max_M*_ is for both high and low affinity binding sites for MABs, and *B*_*max-MH*_ and *B*_*max_ML*_ values are 50% of the total *B*_*max_M*_ as suggested by the two-site model in Owen et al. (2010).

b *B*_*max_H*_ was used as *B*_*max_M*_ for ^11^C-(R)-PK11195 as there is no significant difference between *B*_*max_H*_ and *B*_*max_L*_ for this ligand as shown in Owen et al. (2010).

**Table 2:** In vivo prediction of *K*_1_, *k*_2_ and *BP*_*ND*_ for each radioligand and binding class in normal subjects.

Radioligands	*K*_1_	*k*_2_	*BP*_*ND*_		
HAB	MAB	LAB
^18^F-PBR111	0.25	0.08	0.72	0.44	0.09
^11^C-PBR28	0.53	0.25	3.99	1.96	0.04
^11^C-DPA713	0.48	0.19	1.60	0.94	0.19
^11^C-(R)-PK11195	0.09	0.01	0.20	0.23	0.30

Units for *K*_1_ and *k*_2_ are mL·cm^−3^·min and min^−1^ respectively; *BP*_*ND*_ is unitless.

**Table 3:** %*COV*[*BP*_*ND*_] for all the ligands in normal and diseased states.

Radioligands	Controls			Disease I (50% increase)			Disease II (400% increase)		
HAB	MAB	LAB	HAB	MAB	LAB	HAB	MAB	LAB
^18^F-PBR111	1.47	2.10	7.71	1.19	1.64	5.48	0.91	0.93	1.95
^11^C-PBR28	1.30	1.55	33.1	1.26	1.31	22.43	1.71	1.27	7.57
^11^C-DPA713	1.61	2.16	6.98	1.45	1.75	4.95	1.29	1.26	2.12
^11^C-(R)-PK11195	36.2	32.2	27.3	27.2	25.8	22.7	17.00	16.5	17.0

**Table 4:** Sample size required for within-subject longitudinal studies.

Binding class	50% increase			400% increase		
Mean Δ*BP*_*ND*_	SD Δ*BP*_*ND*_	Sample size	Mean Δ*BP*_*ND*_	SD Δ*BP*_*ND*_	Sample size
*^18^F-PBR111*						
HAB	0.357	0.094	3	2.874	0.726	3
MAB	0.219	0.054	3	1.748	0.428	3
LAB	0.047	0.016	4	0.378	0.096	3

*^11^C-PBR28*						
HAB	1.997	0.503	3	15.88	3.945	3
MAB	0.983	0.239	3	7.759	2.043	3
LAB	0.018	0.023	19	1.496	0.379	3

*^11^C-DPA713*						
HAB	0.769	0.194	3	6.187	1.517	3
MAB	0.460	0.118	3	3.650	0.904	3
LAB	0.091	0.032	4	0.721	0.184	3

*^11^C-(R)-PK11195*						
HAB	0.071	0.109	27	0.619	0.236	4
MAB	0.091	0.115	19	0.701	0.260	4
LAB	0.113	0.129	16	0.934	0.342	4

**Table 5:** Sample size required for between-subjects studies when binding class information is unknown.

Radioligand	50% increase				400% increase			
Mean *BP*_*ND*_disease_ − Mean *BP*_*ND*_control_	SD *BP*_*ND*_control_	SD *BP*_*ND*_disease_	Sample size	Mean *BP*_*ND*_disease_ − Mean *BP*_*ND*_control_	SD *BP*_*ND*_control_	SD *BP*_*ND*_disease_	Sample size
*Caucasians*								
^18^F-PBR111	0.270	0.242	0.362	33	2.187	0.249	1.225	7
^11^C-PBR28	1.396	1.506	2.290	49	11.14	1.507	7.608	8
^11^C-DPA713	0.592	0.543	0.817	35	4.804	0.551	2.732	7
^11^C-(R)-PK11195	0.109	0.101	0.129	29	0.887	0.101	0.384	6

*African Americans*								
^18^F-PBR111	0.286	0.234	0.350	28	2.305	0.242	1.181	4
^11^C-PBR28	1.502	1.460	2.228	40	11.97	1.460	7.396	7
^11^C-DPA713	0.625	0.525	0.793	29	5.080	0.533	2.646	6
^11^C-(R)-PK11195	0.107	0.099	0.126	29	0.870	0.099	0.371	6

*Han Chinese and Japanese*								
^18^F-PBR111	0.353	0.187	0.274	13	2.813	0.194	0.949	5
^11^C-PBR28	1.955	1.082	1.666	14	15.50	1.073	5.609	5
^11^C-DPA713	0.771	0.418	0.637	13	6.244	0.425	2.104	5
^11^C-(R)-PK11195	0.097	0.092	0.117	30	0.815	0.090	0.328	5

**Table 6:** Sample size required for between subject studies when binding class information is known a priori.

Binding class	50% increase				400% increase			
Mean *BP*_*ND*_disease_ − Mean *BP*_*ND*_control_	SD *BP*_*ND*_control_	SD *BP*_*ND*_disease_	Sample size *t*-test	Mean *BP*_*ND*_disease_ − Mean *BP*_*ND*_control_	SD *BP*_*ND*_control_	SD *BP*_*ND*_disease_	Sample size *t*-test
*^18^F-PBR111*								
HAB	0.349	0.182	0.265	10	2.879	0.184	0.906	4
MAB	0.214	0.111	0.164	10	1.762	0.117	0.559	4
LAB	0.044	0.028	0.040	14	0.370	0.025	0.118	4

*^11^C-PBR28*								
HAB	2.021	0.992	1.531	10	15.99	0.979	5.182	4
MAB	0.956	0.498	0.753	11	7.880	0.499	2.501	4
LAB	0.019	0.017	0.021	22	0.149	0.018	0.049	4

*^11^C-(R)-DPA713*								
HAB	0.790	0.397	0.606	10	6.399	0.403	1.999	4
MAB	0.464	0.243	0.358	10	3.813	0.239	1.150	4
LAB	0.093	0.051	0.072	11	0.725	0.048	0.238	4

*^11^C-(R)-PK11195*								
HAB	0.095	0.092	0.116	27	0.810	0.088	0.324	4
MAB	0.119	0.100	0.122	20	0.903	0.100	0.360	4
LAB	0.140	0.117	0.155	21	1.220	0.112	0.474	4
